# Anti-SRP Antibodies and Myocarditis in Systemic Sclerosis Overlap Syndrome with Immune-Mediated Necrotizing Myositis (IMNM)

**DOI:** 10.3390/medicina60111756

**Published:** 2024-10-26

**Authors:** Cristina Alexandru, Anca Donisa, Florin Bobirca, Ana Maria Dascalu, Dan Dumitrescu, Ioan Ancuta, Mihai Bojinca, Ana Maria Balahura, Carmen Manea, Ionela Belaconi, Daniela Anghel, Catalin Dumitrașcu, Catalin Alius, Andreea Cristina Costea, Andrei Marin, Dragos Serban, Anca Bobircă

**Affiliations:** 1 Faculty of Medicine, “Carol Davila” University of Medicine and Pharmacy, 020021 Bucharest, Romaniaionela.belaconi@umfcd.ro (I.B.); dragos.serban@umfcd.ro (D.S.); anca.bobirca@umfcd.ro (A.B.); 2 Internal Medicine and Rheumatology Department, “Dr. Ion Cantacuzino” Clinical Hospital, 011437 Bucharest, Romania; 3 Department of Pneumology, “Marius Nasta” Institute of Pneumology, 010024 Bucharest, Romania; 4 Surgery Department, “Dr. Ion Cantacuzino” Clinical Hospital, 011437 Bucharest, Romania; 5 Ophthalmology Department, Emergency University Hospital Bucharest, 050098 Bucharest, Romania; 6 Fourth General Surgery Department, Emergency University Hospital Bucharest, 050098 Bucharest, Romania; 7 Cardiology Department, Clinical Hospital “Prof. Dr. Th. Burghele”, 061344 Bucharest, Romania; 8 Rheumatology and Internal Medicine Department, “Sfanta Maria” Clinical Hospital Bucharest, 011172 Bucharest, Romania; 9 Internal Medicine Department, “Dr. Carol Davila” Central Military Emergency University Hospital, 010242 Bucharest, Romania; 10 Department of Nephrology, Diaverum Clinic, 900612 Constanta, Romania; 11 Plastic Surgery Department, “Sf. Ioan” Emergency Clinical Hospital, 042122 Bucharest, Romania

**Keywords:** systemic sclerosis, myocarditis, anti-SRP antibodies, necrotizing myositis

## Abstract

Overlap syndrome of systemic sclerosis and idiopathic inflammatory myopathies is an increasingly frequent entity, but the association with immune-mediated necrotizing myositis has rarely been described. While myositis or myopathy may be features of scleroderma, it is imperative to correctly diagnose an overlap syndrome of these two, since it can be considered a different entity with specific management and a worse prognosis. Anti-signal recognition particle (anti-SRP) antibodies target the striated muscle fiber and inhibit myoblast regeneration, resulting in myofiber atrophy and necrosis. Anti-SRP antibodies are specific in immune-mediated necrotizing myopathy characterized by myonecrosis and minimal inflammatory reaction, with proximal muscle weakness and typical extra-muscular manifestation. There are controversial data on the association of cardiac manifestations and the presence of these antibodies, and recent studies cannot prove a significant correlation between the two. Myocarditis is a complication with an unpredictable, potentially severe outcome from heart failure and dilated cardiomyopathy to fatality. It can be difficult to diagnose, and a myocardial biopsy can be problematic in daily practice; thus, most practitioners rely on cardiac magnetic resonance with suggestive images for the correct diagnosis. This paper seeks to address the challenges associated with the diagnosis and treatment of collagen diseases by evaluating the role of anti-SRP antibodies in the pathogenesis of cardiac involvement.

## 1. Introduction

Systemic sclerosis or scleroderma (SSc) is a systemic autoimmune disease, characterized by excessive fibrosis of the skin and internal organs such as the lungs, heart, kidneys, and digestive tract [1]. Idiopathic inflammatory myopathies (IIMs) represent a heterogeneous group of connective tissue diseases characterized by muscular inflammation and extra-muscular damage. The group comprises dermatomyositis, polymyositis, inclusion body myositis, anti-synthetase syndrome, and immune-mediated necrotizing myositis (IMNM) [2]. While myositis or myopathy may be features of scleroderma, it is imperative to correctly diagnose an overlap syndrome of these two, since it can be considered a different entity with specific management and a worse prognosis [3]. Overlap syndrome of scleroderma with idiopathic inflammatory myopathies such as polymyositis or dermatomyositis is more frequent, and usually associated with positive anti-PM/Scl and anti-Ku antibodies [4]. Patients with positive anti-PM/Scl antibodies are more likely to develop myositis, pulmonary interstitial fibrosis, calcinosis, telangiectasia, and dermatomyositis-like skin manifestations.

In contrast, esophageal involvement, pulmonary arterial hypertension, or cardiac involvement are less frequent among these patients [5]. On the other hand, anti-Ku antibodies have been linked to polymyositis–scleroderma overlap since 1981, with patients presenting synovitis, joint contractures, and myositis symptoms but without vascular damage [6]. Patients with SSc are at high risk of developing interstitial lung disease (ILD), a severe complication with great mortality and morbidity; around 30% of cases lead to respiratory failure in the first 5 years after diagnosis [1]. ILD in SSc patients accounts for 17% of SSc-related deaths, with non-specific interstitial pneumonia (NSIP) being the most prevalent subtype on high-resolution computed tomography [HRCT] images [7]. Recent findings have shown that anti-PM/Scl and anti-Ku antibodies are risk factors for severe interstitial lung disease, but relatively modest vascular complications [5]. Another antibody associated with connective tissue diseases is an anti-signal recognition particle (anti-SRP), usually more frequently described in patients with IMNM. Still, these antibodies are also described in several cases of systemic scleroderma or other subtypes of myositis. These antibodies usually come with a specific clinical aspect predominantly proximal muscle pain and dysphagia but chest pain, arthritis, arthralgia, Sicca syndrome, carpal tunnel syndrome, ILD, and skin rash can be present [2,3].

## 2. Immune-Mediated Necrotizing Myositis (INMN)

INMN is a newly described subtype of idiopathic inflammatory myopathy, frequently presenting with anti-signal recognition particle and anti-3-hydroxy-3-methylglutaryl-coa reductase (anti-HMGCR) antibody positivity [8]. Patients with IMNM have proximal muscle involvement, with a muscle biopsy showing myofiber necrosis with a paucity of lymphocyte infiltrates [9]. Cases of scleroderma overlapping with myopathies with muscle necrosis and damage of small vessels with a “pipe-stern” appearance, with positive anti-SRP antibodies have been described less often [10]. Anti-SRP antibodies are more frequently associated with pain in the proximal muscles and diffuse myonecrosis in the striated muscles, leading to myalgia, dysphagia, and dyspnea with respiratory failure due to fatigue of the respiratory muscles [11]. Recent data support a new theory suggesting that the overlap between scleroderma and myositis represents a distinct disease known as scleromyositis, characterized by specific clinical and immunological features. However, despite a high frequency of IMNM characteristics at muscle biopsy in scleromyositis patients, these patterns are shown to be associated with anti-Ku, anti-PM/SCL, and anti-U1-RNP autoantibodies, rather than with anti-SRP or anti-HMGCR antibodies [12].

Cardiac manifestations in idiopathic inflammatory myopathies and SSc are frequent and can range from subclinical myocardial damage, often identified at cardiac magnetic resonance [CMR], to arrhythmia and heart failure [13]. The cardiac symptoms and their progression are not well defined, and additional studies are needed to establish a specific set of biomarkers in these patients and better characterize the subtypes with a worse outcome. In small studies, anti-SRP antibodies are associated with more frequent myocardial involvement; however, this association was not identified in large studies [14].

## 3. Cardiac Involvement in Systemic Scleroderma

Cardiac complications in systemic scleroderma are prevalent and frequently underdiagnosed. These complications may arise directly from scleroderma’s impact on the myocardium or indirectly from associated conditions such as pulmonary arterial hypertension, interstitial lung disease, or renal crisis. Fibrosis, inflammation, and vascular alterations can affect all cardiac structures, occurring in approximately 80% of systemic scleroderma patients, although they often present without any cardiac symptoms [15]. The direct effects of the myocardium are represented by microvascular coronary artery disease, cardiac fibrosis, myocarditis, systolic or diastolic dysfunctions of the right or left ventricle, conduction, and rhythm anomalies, even with the involvement of the pericardium [16,17]. Cardiac complications, especially arrhythmias and congestive heart failure, represent the second leading cause of death in patients with systemic scleroderma, while in patients with IIM, cardiac complications are responsible for 5% of deaths [18,19]. Additionally, muscle myopathy is associated with a heightened risk of cardiac complications and increased mortality [12]. Microvascular damage and immune dysfunction are the primary pathological mechanisms leading to the accumulation of collagen deposits, which can result in hyperplasia of intramural arteries and intermittent ischemia. Necrotic areas that develop within the myocardium are subsequently replaced by patchy myocardial fibrosis. This fibrotic pattern, regarded as a hallmark of scleroderma of the myocardium, can affect both ventricles and occurs independently of the coronary artery distribution [16]. Cardiac magnetic resonance imaging is the most effective method for detecting cardiac involvement in connective tissue diseases, revealing that heart involvement in patients with SSc is more common than previously recognized [20]. Diastolic dysfunction appears to be common in systemic scleroderma and may affect either ventricle. Impaired ventricular filling is indicative of a fibrotic and rigid ventricle, which can lead to atrial dilatation [20]. Frequent cardiac manifestations are arrhythmias, congestive heart disease, pericarditis, and myocarditis, but primary heart involvement is difficult to establish in patients with SSc [15]. Changes in rhythm, such as supraventricular or ventricular tachycardias can be responsible for sudden death in some patients. Supraventricular arrhythmias are the most frequently observed abnormalities during 24 h Holter monitoring, whereas ventricular arrhythmias, although less common, may present as polymorphic, often characterized by premature contractions [16].

Because of the similarities between the myocardium and skeletal muscles, the heart can also be affected by myositis as part of the same inflammatory process [21]. Although significant cardiac involvement is relatively uncommon, it remains a critical cause of death. This is particularly important in subclinical cases, where proinflammatory cytokines alongside traditional cardiovascular risk factors play a crucial role in diastolic dysfunction [22]. Diastolic dysfunction of the left ventricle is the most common type of cardiac damage in myositis. However, case reports have also documented coronary damage, including angina pectoris, and myocardial infarction [13]. In contrast, subclinical damage appears to be more common. This includes changes such as supraventricular or ventricular arrhythmias, bundle branch blocks, atrioventricular blocks, PR interval prolongations, ventricular premature beats, and abnormal Q waves or ST-T changes, usually detected on the ECG of a patient with myositis [23]. Regarding IIM, the pathogenesis of cardiac changes includes the inflammatory infiltration of the conduction system, with the replacement of cardiomyocytes from it, with fibrosis, and vasculitis of the small vessels that irrigate the myocardium, along with the infiltration of the myocardium itself [24].

The frequency of cardiac involvement in IIM can range from 9.3% to 61%, with arrhythmia being the most common [19,25,26]. Diederichsen et al. showed that cardiac manifestations are more common in newly diagnosed patients naïve to treatment than in controls, proving the negative effect of an inflammatory burden on cardiac function [27].

A recent review which included 29 studies showed the cardiac implications in IIM patients, demonstrating that despite the heterogeneity of IIM, some cardiac patterns can be defined [13]. On the other hand, cardiac changes in patients with myositis can be associated with corticosteroid therapy, making it extremely challenging to distinguish whether these changes are due to the corticosteroids [CSs], or the disease itself. In IIM, corticosteroids are still a reliable therapy in addition to immunosuppressives. CSs are well-known risk factors for hypertension, and increased dosages for a prolonged time are associated with heart failure and decreased ejection fraction [28].

Myocarditis can be difficult to diagnose, being defined as myocardial inflammation on endomyocardial biopsy with specific characteristics [29]. Currently, a myocardial biopsy is rare, and most practitioners rely on cardiac magnetic resonance [CMR] with suggestive images for the correct diagnosis [30]. Myocardial alterations on CMR in IIM patients without any cardiac manifestation are frequent [31]. Myocarditis in IIM can be the first presentation or developed during the follow-up; its prevalence is not well studied, but it can vary around 2.6–3.4%, with a substantial increase at a histopathological examination of 38% of cases [19,32]. The evolution can be severe, from fatality to heart failure or dilated cardiomyopathy. In a study on anti-synthetase syndrome patients with myocarditis, the authors showed a strong correlation between myositis activity and the development of myocarditis, but there was no definition of negative prognostic factors for these patients [32].

Heart involvement is more frequent in overlap syndrome of SSc and myositis [3]. A recent case report by Garcia et al. presented an overlap syndrome of systemic sclerosis and polymyositis with anti-SCL-70 and anti-PL/SCL-100 antibodies with lymphocytic myocarditis responding to cyclophosphamide monthly pulses [33].

## 4. The Role of Anti-SRP Antibodies

Anti-SRP antibodies are antibodies specific to immune-mediated necrotizing myopathy characterized by myonecrosis and minimal inflammatory reaction. The actual mechanism of anti-SRP pathogenesis has yet to be established. These antibodies target the striated muscle fiber and inhibit myoblast regeneration, resulting in myofiber atrophy and necrosis [8]. Furthermore, in vivo studies revealed that transferring anti-SRP antibodies from patients to mice caused muscular deficit via a complement-related mechanism [34]. There is currently contradictory research about the associations between serum creatine kinase (CK) and anti-SRP antibodies [35,36]. Reversely, antibody serum concentrations in subtypes of IMNM with anti-HMGCR positivity are strongly correlated with CK levels and high antibody titer, suggesting the severity of muscle involvement [37]. Another distinguishing feature of anti-SRP positive is the duration of muscle involvement when compared to other types of myositis. One study found a remission of just 25% after one year and 50% at four years, albeit with a persistently elevated CK level [38].

**Figure 1:**
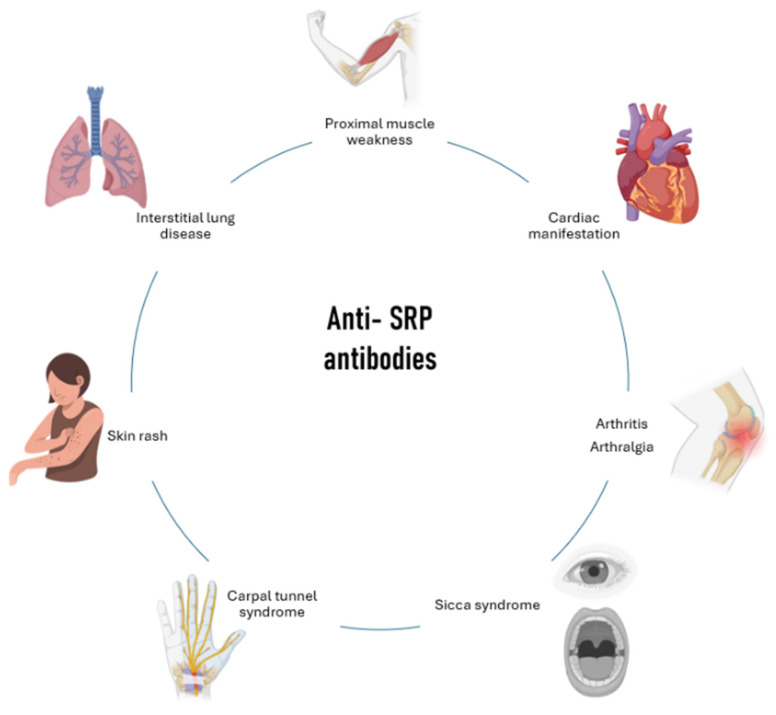
Clinical manifestations associated with anti-signal recognition particle (anti-SRP) antibodies.

Anti-SRP IMNM is characterized by proximal muscle weakness, which can be associated with dyspnea and dysphagia. Different studies have defined a distinct extra-muscular phenotype in the presence of anti-SRP antibodies, including chest pain, arthritis, arthralgia, Sicca syndrome, carpal tunnel syndrome, ILD, and skin rash (Figure 1) [11].

Cardiac involvement in the presence of these antibodies is described, with arrhythmias, cardiomyopathy, and gradual evolution toward heart failure. However, data on cardiac involvement come from small cohort studies, clinical cases, or studies conducted on larger cohorts but with a different object, with cardiac involvement only appearing in the description section. Thus, in this paper, we gather all studies on this subject, after a literature search on two medical databases (PubMed/Medline and Google Scholar) using the terms “anti-SRP”, “anti-signal recognition particle antibodies”, “IMNM”, “immune-mediated necrotizing myositis” and “cardiac involvement”, “myocardial involvement”. The results are structured in Table 1.

In a recent study, following thirty-nine patients with anti-SPR-IMNM, no cardiac involvement was reported in the entire cohort, although the focus of the study was more on muscle strength rather than extra-muscular manifestation [38]. However, a larger study by Suzuki et al. following one hundred patients with anti-SRP IMNM showed that cardiac muscle interest was a rare manifestation, with only two subjects describing it [35].

Kao et al. followed patients with anti-SRP antibodies and showed that among 790 SSc patients, only 2 had anti-SRP positivity and developed muscle symptoms after a median of 3.5 years. Moreover, among nineteen anti-SRP patients in the cohort (diagnosed with polymyositis, SSc, and anti-synthetase syndrome) cardiac manifestations occurred in 15.8% (three patients), while ILD in 31.57% (six patients) [39]. On the other hand, Hengstman et al. analyzed a large cohort, identifying only five patients diagnosed with polymyositis or dermatomyositis with anti-SRP positivity with severe myalgia and arthritis and resistance to treatment, but none had cardiac manifestations [40]. Later on, they compared a group of anti-SRP patients with polymyositis/dermatomyositis patients with negative anti-SRP, and their results showed no differences in frequency in electrocardiogram abnormalities. Although they reported cardiac anomalies in more than half of patients [clinical symptomatology and electrocardiogram alteration], their results were influenced by cardiovascular factors and concomitant cardiac diseases or ILD [41]. Thus, a well-designed study is needed to clarify the association between anti-SRP antibodies and cardiac manifestations.

One recent study by Bandeira et al. analyzed the cardiac involvement in IIM patients. The results showed that in comparison with patients without cardiac involvement, anti-SRP antibodies are the most commonly identified antibodies (76.9% vs. 31.5%, *p* = 0.001). Moreover, in patients with cardiac manifestation, there was also more frequently reported lung and esophageal involvement, suggesting that patients with systemic manifestations are also more predisposed to cardiac symptomatology and a thorough evaluation is needed [42].

One case report presented severe cardiomyopathy in anti-SRP and anti-MDA-5 IMNM, with progression after immunosuppressive therapy, despite improvement of the clinical myopathy, which needed heart transplantation, supporting the data that this subset of antibodies is a negative prognostic factor [43,44]. Several case reports and case series present a severe course of disease in IMNM patients with anti-SRP positivity presenting with cardiac complications [45,46,47,48,49,50].

**Table 1:** Literature search on anti-SRP antibodies and cardiac involvement.

Study	Year	Cohort	Cardiac Involvement
Targoff et al. [49]	1990	12 anti-SRP PM/DM patients	4/12
Hengstman et al. [40]	2002	5 anti-SRP PM/DM patients	0
Miller et al. [50]	2002	7 anti-SRP patients	0
Kao et al. [39]	2004	19 anti-SRP patients (16 PM, 2 SSc, 1 aSS)	3/19
Hengstman et al. [41]	2006	23 anti-SRP patients	6/23
Suzuki et al. [35]	2015	100 anti-SRP patients	2/100
Pinal-Fernandez et al. [38]	2017	37 anti-SRP patients	0
Bandeira et al. [42]	2023	12 anti-SRP patients	3/12

## 5. Clinical and Paraclinical Presentation

**Figure 2:**
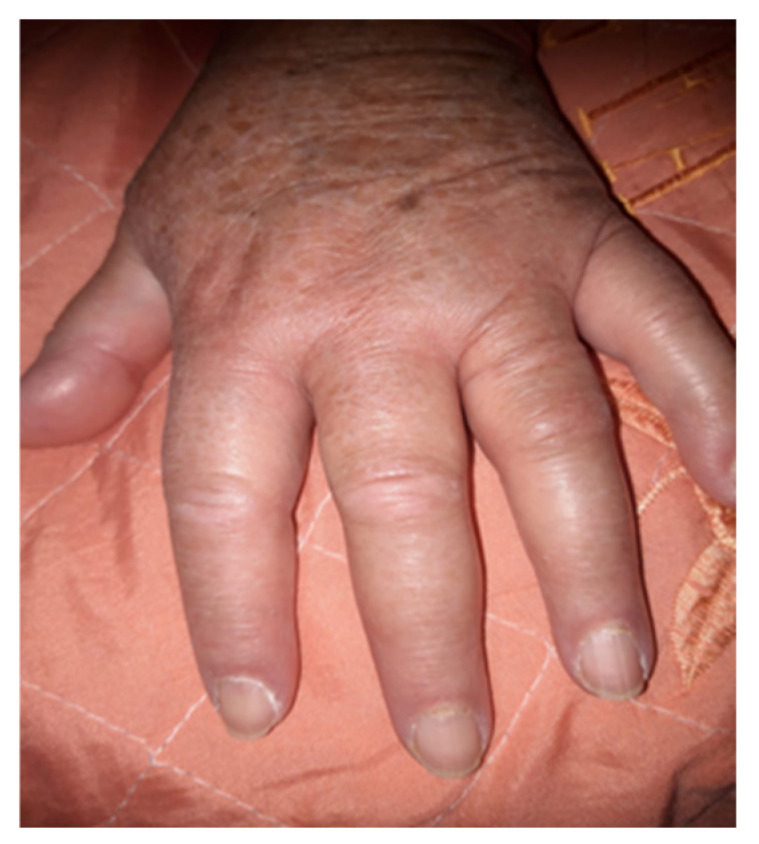
Puffy fingers at the first presentation (September 2021).

A 74-year-old female, with a history of type 2 diabetes mellitus and arterial hypertension, diagnosed with systemic scleroderma in May 2021, was referred to the rheumatology department in September 2021 to establish the magnitude of organ involvement and to decide the appropriate therapeutic approach. She described Raynaud’s phenomenon, arthritis, and mild dyspnea. The physical examination revealed loss of wrinkling, telangiectasias, microstomia, sclerodactyly with puffy fingers achieving a 6/51 Rodnan Score, and velcro bibasilar crackles by lung auscultation (Figure 2).

**Figure 3:**
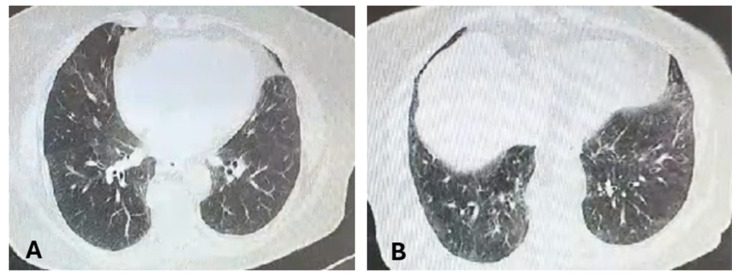
Chest CT September 2021. (**A**) Unsystematized bilateral ground-glass pulmonary infiltrates; (**B**) bilateral basal ground-glass pulmonary infiltrates.

Mild anemia (hemoglobin 10.9 g/dL; normal value 11.7–15 g/dL) and high positivity for anti-topoisomerase I antibodies were shown by the blood test evaluation and a late sclerodermic pattern was described at nail video-capillaroscopy. The pulmonary evaluation included chest HRCT [bilateral ground-glass pulmonary infiltrates-NSIP—Figure 3] and spirometry [lung volumes were normal with a slightly decreased DLco (68% of predicted value)], while echocardiography found pulmonary hypertension [43 mmHg] with normal ejection fraction. Throughout the upper endoscopy examination, no indications of esophageal involvement were observed.

**Figure 4:**
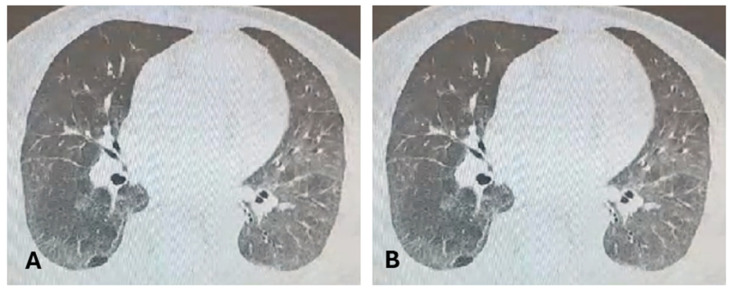
Chest CT September 2022. (**A**) NSIP-like appearance; (**B**) basal aspect of NSIP-like bilateral infiltrates.

Based on the ACR/EULAR criteria [16/28 points] [14], systemic sclerosis with cutaneous, pulmonary, and vascular involvement was diagnosed, and the patient started immunosuppressive treatment. Initially, Mycophenolate mofetil (MMF) was started at 2 g daily, but the patient described intense nausea, abdominal pain and diarrhea. Hence, due to the adverse events associated, MMF was tapered to 1 g daily, together with proton pump inhibitors and calcium channel blockers (CCBs). We decided to switch to intravenous (iv) cyclophosphamide (800 mg monthly for six months) in May 2022 due to the clinical progression (significant progression of the skin fibrosis with a Rodnan score of 14/51) and lung damage with a decrease in DLco (54% of predicted value) and progress of interstitial fibrosis at chest HRCT. It was suggested to start antifibrotic Nintedanib therapy; nevertheless, she did not meet the initiation criteria of the national protocol. The results of the lab tests showed a rising pattern in the levels of inflammatory syndrome (maximum ESR = 59 mm/h; CRP = 33 mg) and creatine kinase [two to three times higher than normal values], and further lung function modification, as indicated by a 50% drop in DLco with stable chest HRCT (Figure 4). A myositis-specific antibody profile was assessed in light of a potential overlapping myositis, and the results indicated that anti-SRP antibodies were present.

**Figure 5:**
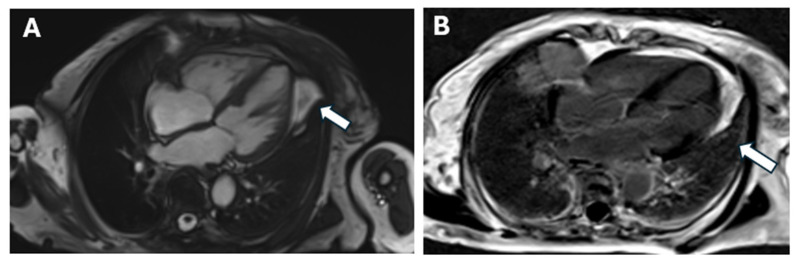
Cardiac MRI. (**A**) Aspect of chronic myocarditis; arrow—the lateral wall of the left ventricle with hypokinesia; no edema; (**B**) LGE PSIR; arrow—Gadolinium accumulation area in the lateral wall of the left ventricle.

The patient experienced a sudden hypertensive spike in November 2022 during the sixth and last cycle of cyclophosphamide. The patient also experienced dyspnea and tachypnea, as well as an 80% desaturation under five liters of oxygen supplementation. The ECG revealed sinus tachycardia and serum troponins were slightly elevated. Importantly, NT-proBNP increased to 35,000 pg/mL. Due to a suspicion of a myocardial infarction other than ST-segment elevation, the patient was referred to the cardiology department. The right ventricle was slightly dilated up to the upper limit of normal values, with normal longitudinal systolic function, mild biatrial dilatation, aortic sclerosis, and possible pulmonary hypertension. Firstly, an echocardiography was performed and it showed a non-dilated left ventricle with moderate global and segmental systolic dysfunction, and hypokinesis of the circumferential apex, inferior wall, and interventricular septum, with intramyocardial hyperechoic areas. Coronary angiography was advised following this incident, but the patient refused. Cardiac magnetic resonance imaging was performed, and the results showed non-dilated ventricles with preserved global systolic function, segmental kinetic disorders, hypokinesis of the left ventricle’s lateral wall, indexed myocardial mass within normal limits, replacement myocardial fibrosis with myocarditis pattern, biatrial dilatation, ascending aorta ectasia, and absence of pulmonary artery dilatation (Figure 5).

No immunosuppressive treatment was administered until January 2023 (Figure 5). A skin and muscle biopsy was performed, after a reasonable disclosure [51] in light of the patient’s fatigue, the cardiac episode in November 2022, the elevated CK and CK-MB values in recent months, positive anti-SRP antibodies, muscle atrophy, and the suspicion of immune necrotizing myopathy overlapping systemic sclerosis or maybe a scleromyositis. The histopathological examination indicated a non-specific myopathic appearance with morphological elements consistent with a necrotizing myopathy in the defined clinical-paraclinical context (positive anti-SRP antibodies). There was also a notable variation in the shape of the muscle fibers, with atrophic fibers varying in length and polygonal form, moderate adipose metaplasia, minimal interstitial fibrosis, and rare necrotic fibers. We concluded that the overlapping hypothesis was the most likely to be accurate; therefore, we started the immunosuppressive therapy with Azathioprine 100 mg daily and moderate doses of Prednisone, tapering to a low level.

**Figure 6:**
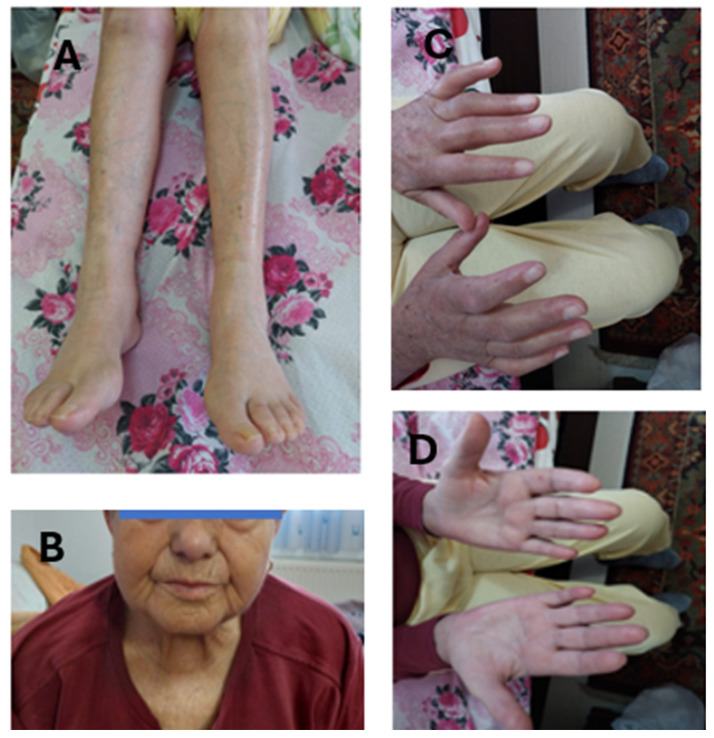
Clinical aspect of the patient in January 2023: (**A**) patient’s legs with no edema and hair loss; (**B**) patient’s hands- sclerodactyly; (**C**) patient’s face showing microstomia; (**D**) patient’s hand-telangiectasias.

**Figure 7:**
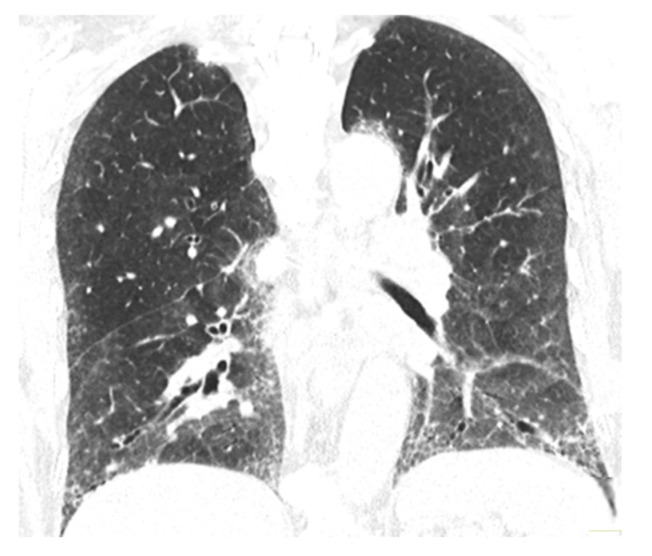
Chest CT September 2023. Bilateral infiltrates slightly changed compared to September 2022; reconstruction in coronal plane.

Although the patient’s condition was clinically stable in September 2023, on Azathioprine and 5 mg Prednisone, the DLco decreased by 40% of what was expected, the chest CT scan revealed interstitial changes and slightly altered micronodulations from September 2022 (Figure 6 and Figure 7), and the blood tests continued to reveal a slight anemia and low inflammatory syndrome, so the patient received a single dose of Tocilizumab (400 mg iv administration) with no possibilities to continuing to administrate the biological therapy.

The lung CT scan performed in February 2024 revealed a typical NSIP-like appearance of fibrosis as well as functional deterioration with a DLco of 31% from the predicted one. Thus, the pneumologist decided to start the antifibrotic medication-nintedanib 200 mg/day, and the patient kept taking the specific cardiac drugs, with recurrent interdisciplinary evaluation and favorable cardiac evolution.

## 6. Discussion

The anti-SRP antibodies are very uncommon among SSc patients, with a prevalence of around 0.7%, and even lower in overlap syndrome [52]. IMNM is a less prevalent subtype of IIM, accounting for 18–39% of cases, with an average age of onset of 40–50 years, contrary to our case with an elderly disease onset [8].

**Figure 8:**
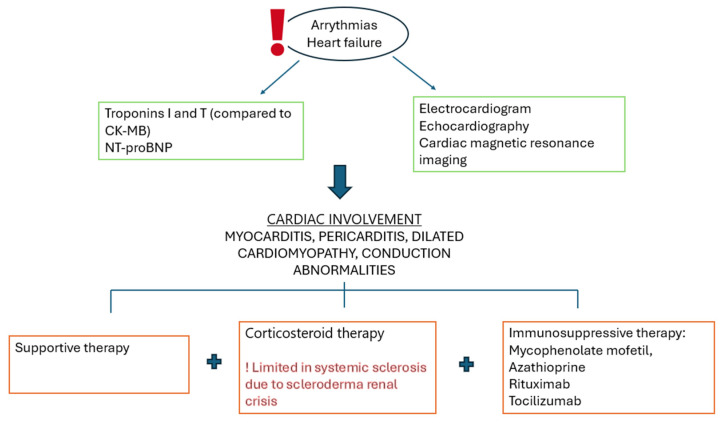
General management steps of cardiac involvement in anti-SRP patients.

There are several studies describing cardiac complications in SSc and IMNM, and although in the past there was a strong correlation between anti-SRP antibodies and cardiac manifestations, our research has not proved this correlation (Table 1). This theory needs to be validated in large, well-controlled studies, but a major concern still remains, and supplementary cardiac investigations are recommended in these patients. Such cases are difficult to diagnose and there is no general protocol on the management in these cases. In Figure 8, we try to summarize the diagnosis and treatment steps.

Other antibodies that are more frequently associated with cardiac manifestation in IIM patients are anti Jo1 anti bodies (33.3%) and anti SSA/SSB antibodies (33.3%), but heart involvement in connective tissue diseases is still an undervalued field and in great need of further studies [42].

The most frequent cardiac manifestation in SSc and in IIM are arrhythmias, and our patients develop tachycardia as a symptom of the underlying myocarditis [15,26]. In terms of age interfering with the disease characteristics, younger age is associated with a more difficult-to-treat form of IMNM in anti-SRP-positive patients, in contrast with other subtypes of IIM [35,38]. In our case, although the muscle injury was minor by the literature, cardiac complications such as myocarditis provided diagnostic and therapeutic challenges. A similar pattern was described by Ma et al., showing progressive cardiac involvement, regardless of the good clinical response under immunotherapy [43]. The early identification of cardiac involvement can be achieved by measuring troponins I and T, which rapidly detect myocardial injury, especially troponin I in acute coronary damage, with troponin T also influenced by skeletal muscle damage; both are more specific compared to CK-MB measurement. Another relevant tool in the case of these individuals is NTproBNP, which can identify hemodynamic and structural abnormalities in their preclinical period [2]. Nailfold video-capillaroscopy is an essential diagnostic tool in SSc patients with Raynaud’s phenomenon, with a specific pattern, and it can have a prognostic factor too. In IIM, although alteration is identified, there is no specificity for myositis, nor for other autoimmune diseases [53,54].

We experienced challenges in establishing our patient’s accurate diagnosis; although initially SSc was a facile diagnosis, when the patient later developed muscle involvement, asthenia, muscle atrophy, muscle cytolysis, and cardiac involvement equivalent to myocarditis, with anti-SRP positivity, a series of theories appeared: to integrate cardiac and muscle involvement in the context of SSc, to reconsider the diagnosis as scleromyositis (a new diagnostic entity in rheumatology that is under-explored or neglected), or to conclude that we were faced with an overlapping syndrome of SSc and IMNM. We could argue that the following subpoints support the confirmation of scleromyositis in our patient: elevated muscle enzymes, more frequent cardiac involvement [myocarditis], muscular manifestations, and rapid progression of pulmonary involvement. The classical hallmarks of muscle biopsy, which include vasculopathy and fibrotic lesions without necrosis and inflammation, and the absence of anti-Pm/Scl antibodies work against the diagnosis [12]. But we concluded that the overlapping syndrome of SS and IMNM is the appropriate diagnosis sustained by the muscular biopsy findings—a necrotizing myopathy in the context of anti-SRP positivity and the severity of organ involvement, such as cardiac pulmonary hypertension and myocarditis, ILD [55].

Regarding the acute onset of cardiac manifestation, we took into account the possible adverse effects of Cyclophosphamide (CYC), although Cyclophosphamide has been for years the standard of treatment for systemic sclerosis, particularly in cases of heart involvement [myocarditis] [56]. Administered intravenously or orally in SSc, CYC has some potential risks including hemorrhagic cystitis, alopecia, vomiting, diarrhea, and skin and bladder cancer, as well as possibly irreversible infertility; cardiac side effects are not as prevalent [57,58]. CyC is a strong alkylating medication used in oncology. Many cardiac adverse events associated with its metabolites have been reported in the literature. They are divided into two categories: irreversible, which is heart failure caused by the death of cardiomyocytes as a result of oxidative stress or the platelets’ reactivity-arachidonic pathway, and reversible, which is cardiac remodeling due to the impairment of cardiomyocytes’ function as a result of proinflammatory cytokines [59]. Such side effects are not typically reported in SSc, as a consequence of the lowest dosages used for ILD, but when autologous stem cell transplantation (ASCT) was used more often for SS patients, the dosage of CYC had to be increased, which resulted in the automatic onset of secondary cardiac dysfunction. It is important to discuss the case of a patient with SSc with ILD who had no prior cardiac involvement and no cardiac risk, and was treated with CYC 12g IV over four days for ASCT. At twelve hours after ASCT, the patient developed acute heart failure (BNP of 7136 ng/L and widespread hypokinesia with an LVEFo10%). The patient passed away on day nine following ASCT despite receiving intense supportive care. A necropsy revealed significant pericarditis, intracavitary thrombus, and myonecrosis without any signs of inflammation, fibrosis, or hypertrophy, leading the examiners to conclude that the cause of death was acute cardiotoxicity caused by CYC [60]. Although acute CYC cardiotoxicity is potentially lethal, it currently appears to be quite rare, mostly due to pre-existing cardiovascular involvement not being given proper consideration. Consequently, extensive pre-transplant disease-specific screening is essential to prevent underlying undiagnosed SSc-related cardiopathy because this kind of therapeutic approach will be the future medicine for our SSc patients with severe organ involvement [61].

Considering our patient’s pre-existing cardiovascular risk factors and cardiac damage in the context of scleroderma, the concept of cumulative cardiac toxicity of CYC could be brought into consideration (possible pulmonary hypertension). Evaluation using a CMR of the acute event revealed alterations suggesting myocarditis that are more likely due to collagen disease but could also be induced by CYC [4.5 g cumulative dosage over 6 months].

There is no formal recommendation for the treatment of cardiac involvement either in scleroderma or in myositis. Usually, the treatment is supportive, depending on the type of cardiac damage. In some studies, a slight amelioration of cardiac output was observed during immunosuppressive treatment, especially in combination with corticosteroids, but with high precaution due to the high risk of scleroderma renal crisis [62]. There have been cases of patients with dermatomyositis and Raynaud’s with ECG changes such as multifocal atrial tachycardia, ventricular premature beats, or blocks, changes that resolved after treatment with Rituximab and corticosteroids [63]. Other patients with myocardial hypokinesia showed normalization in 6 months after treatment with CS and immunosuppressants [64]. Some patients with myositis may present Prinzmetal vasospastic angina, especially in those with Raynaud’s syndrome, which has been successfully treated with high-dose BCC [65]. Due to a lack of cases, treatment in anti-SRP IMNM is not systematically studied, and the efficacity of immunosuppressive treatment is coming from case reports and small cohort studies. A small study showed a favorable response to Rituximab in 76% of cases (13/17), but the duration of the good response is yet to be established [38]. Rituximab has shown promise in the treatment of cardiac and pulmonary complications associated with collagen diseases; nevertheless, there is also evidence of the involvement of tocilizumab [TCZ] in these manifestations. Several Asian studies describe the beneficial effects of TCZ in managing cardiac involvement among patients with SS who have previously received other immunosuppressive treatments, including Rituximab. Following TCZ treatment, the patients’ MRI and ECG showed improvement, probably as a result of IL6 inhibition blocking the inflammatory and fibrotic process, which involves myocardial remodeling [66,67]. Starting from the data indicating the use of TCZ in COVID-19 myocardial disease, we also considered that, in the instance of our patient, a single intravenous dosage of 8 mg/kg may have additional benefits for long-term cardiac outcomes [68].

ILD is a common manifestation in SSc, especially in cases with diffuse fibrosis and anti-Scl 70 antibodies. On the other hand, ILD is associated with anti-SRP IMNM in about 20% of cases [11]. Despite treatment with cyclophosphamide 6 pulses, followed by maintenance therapy with azathioprine, pulmonary fibrosis progressed and nintedanib was initiated.

This paper has several limitations. Firstly, this analysis is based on a single clinical case, and conducting studies on larger cohorts would be advantageous for deriving more definitive conclusions regarding cardiac involvement and the presence of anti-SRP antibodies. Another limitation is the scarcity of data available in the literature, as most insights are derived from case reports and small case series. Additionally, studies involving larger patient populations do not primarily focus on evaluating cardiac manifestations in these patients, which introduces a potential bias due to the study design. 

## 7. Conclusions

In conclusion, this is a case that brings several challenges for both diagnosis and treatment, which could be used as a guide for possible warning outcomes related to antibody seropositivity. Cardiac involvement in connective tissue diseases presents a significant diagnostic challenge, particularly in cases of acute heart failure, which necessitates rapid diagnosis and prompt therapeutic intervention due to its association with high mortality rates in these patients. The current literature lacks sufficient evidence to definitively establish the relationship between anti-SRP antibodies and cardiac dysfunction, highlighting the need for large-scale, rigorously designed studies. While the use of immunosuppressive therapies has shown increasing efficacy in managing pulmonary complications in systemic sclerosis and idiopathic inflammatory myopathies (IIMs), treatment strategies for cardiac involvement remain underdeveloped. This area is complicated by the need to account for both traditional cardiovascular risk factors, which may limit immunosuppressant options, and the potential adverse effects of these therapies [5,35].

## Data Availability

No new data were created.

## References

[1] Kuwana M., Gil-Vila A., Selva-O’Callaghan A. (2021). Role of autoantibodies in the diagnosis and prognosis of interstitial lung disease in autoimmune rheumatic disorders. Ther. Adv. Musculoskelet. Dis..

[2] Chen F., Peng Y., Chen M. (2018). Diagnostic approach to cardiac involvement in idiopathic inflammatory myopathies: A strategy combining cardiac troponin I but not T assay with other methods. Int. Heart J. Int. Heart J. Assoc..

[3] Ranque B., Bérezné A., Le-Guern V., Pagnoux C., Allanore Y., Launay D., Hachulla E., Authier F.J., Gherardi R., Kahan A. (2010). Myopathies related to systemic sclerosis: A case-control study of associated clinical and immunological features. Scand. J. Rheumatol..

[4] Mahler M., Raijmakers R. (2007). Novel aspects of autoantibodies to the PM/Scl complex: Clinical, genetic and diagnostic insights. Autoimmun. Rev..

[5] Cavazzana I., Vojinovic T., Airo P., Fredi M., Ceribelli A., Pedretti E., Lazzaroni M.G., Garrafa E., Franceschini F. (2022). Systemic Sclerosis-Specific Antibodies: Novel and Classical Biomarkers. Clin. Rev. Allergy Immunol..

[6] Mimori T., Akizuki M., Yamagata H., Inada S., Yoshida S., Homma M. (1981). Characterization of a high molecular weight acidic nuclear protein recognized by autoantibodies in sera from patients with polymyositis-scleroderma overlap. J. Clin. Investig..

[7] Elhai M., Meune C., Boubaya M., Avouac J., Hachulla E., Balbir-Gurman A., Riemekasten G., Airò P., Joven B., Vettori S. (2017). Mapping and predicting mortality from systemic sclerosis. Ann. Rheum. Dis..

[8] Watanabe Y., Uruha A., Suzuki S., Nakahara J., Hamanaka K., Takayama K., Suzuki N., Nishino I. (2016). Clinical features and prognosis in anti-SRP and anti-HMGCR necrotizing myopathy. J. Neurol. Neurosurg. Psychiatry.

[9] Anquetil C., Boyer O., Wesner N., Benveniste O., Allenbach Y. (2019). Myositis-specific autoantibodies, a cornerstone in immune-mediated necrotizing myopathy. Autoimmun. Rev..

[10] Pope J.E. (2002). Scleroderma overlap syndromes. Curr. Opin. Rheumatol..

[11] Ma X., Bu B.T. (2022). Anti-SRP immune-mediated necrotizing myopathy: A critical review of current concepts. Front. Immunol..

[12] Giannini M., Ellezam B., Leclair V., Lefebvre F., Troyanov Y., Hudson M., Senécal J.L., Geny B., Landon-Cardinal O., Meyer A. (2023). Scleromyositis: A distinct novel entity within the systemic sclerosis and autoimmune myositis spectrum. Implications for care and pathogenesis. Front. Immunol..

[13] Fairley J.L., Wicks I., Peters S., Day J. (2021). Defining cardiac involvement in idiopathic inflammatory myopathies: A systematic review. Rheumatology.

[14] Allenbach Y., Benveniste O. (2018). Peculiar clinicopathological features of immune-mediated necrotizing myopathies. Curr. Opin. Rheumatol..

[15] Bruni C., Ross L. (2021). Cardiac involvement in systemic sclerosis: Getting to the heart of the matter. Best Pract. Res. Clin. Rheumatol..

[16] Ross L., Costello B., Brown Z., Hansen D., Lindqvist A., Stevens W., Burns A., Prior D., Nikpour M., La Gerche A. (2022). Myocardial fibrosis and arrhythmic burden in systemic sclerosis. Rheumatology.

[17] Glynn P., Hale S., Hussain T., Freed B.H. (2022). Cardiovascular Imaging for Systemic Sclerosis Monitoring and Management. Front. Cardiovasc. Med..

[18] Bairkdar M., Dong Z., Andell P., Hesselstrand R., Holmqvist M. (2024). Arrhythmia in patients with systemic sclerosis: Incidence, risk factors and impact on mortality in a Swedish register-based study. RMD Open.

[19] Gupta R., Wayangankar S.A., Targoff I.N., Hennebry T.A. (2011). Clinical cardiac involvement in idiopathic inflammatory myopathies: A systematic review. Int. J. Cardiol..

[20] Giucă A., Gegenava T., Mihai C.M., Jurcuţ C., Săftoiu A., Gȋrniţă D.M., Popescu B.A., Ajmone Marsan N., Jurcuț R. (2022). Sclerodermic Cardiomyopathy-A State-of-the-Art Review. Diagnostics.

[21] Myhr K.A., Pecini R. (2020). Management of Myocarditis in Myositis: Diagnosis and Treatment. Curr. Rheumatol. Rep..

[22] Schwartz T., Diederichsen L.P., Lundberg I.E., Sjaastad I., Sanner H. (2016). Cardiac involvement in adult and juvenile idiopathic inflammatory myopathies. RMD Open.

[23] Lundberg I.E. (2006). The heart in dermatomyositis and polymyositis. Rheumatology.

[24] Leone O., Veinot J.P., Angelini A., Baandrup U.T., Basso C., Berry G., Bruneval P., Burke M., Butany J., Calabrese F. (2012). 2011 Consensus statement on endomyocardial biopsy from the Association for European Cardiovascular Pathology and the Society for Cardiovascular Pathology. Cardiovasc. Pathol..

[25] Dankó K., Ponyi A., Constantin T., Borgulya G., Szegedi G. (2004). Long-Term Survival of Patients With Idiopathic Inflammatory Myopathies According to Clinical Features. Medicine.

[26] Kassardjian C.D., Lennon V.A., Alfugham N.B., Mahler M., Milone M. (2015). Clinical Features and Treatment Outcomes of Necrotizing Autoimmune Myopathy. JAMA Neurol..

[27] Diederichsen L.P., Simonsen J.A., Diederichsen A.C.P., Kim W.Y., Hvidsten S., Hougaard M., Junker P., Lundberg I.E., Petersen H., Hansen E.S. (2015). Cardiac abnormalities assessed by non-invasive techniques in patients with newly diagnosed idiopathic inflammatory myopathies. Clin. Exp. Rheumatol..

[28] Adamczak D.M., Oduah M.T., Kiebalo T., Nartowicz S., Bęben M., Pochylski M., Ciepłucha A., Gwizdała A., Lesiak M., Straburzyńska-Migaj E. (2020). Heart Failure with Preserved Ejection Fraction—A Concise Review. Curr. Cardiol. Rep..

[29] Al-Akchar M., Shams P., Kiel J. (2017). Acute Myocarditis.

[30] Bernhard B., Schnyder A., Garachemani D., Fischer K., Tanner G., Safarkhanlo Y., Stark A.W., Schütze J., Pavlicek-Bahlo M., Greulich S. (2023). Prognostic Value of Right Ventricular Function in Patients with Suspected Myocarditis Undergoing Cardiac Magnetic Resonance. JACC Cardiovasc. Imaging.

[31] Khoo T., Stokes M.B., Teo K., Proudman S., Basnayake S., Sanders P., Limaye V. (2019). Cardiac involvement in idiopathic inflammatory myopathies detected by cardiac magnetic resonance imaging. Clin. Rheumatol..

[32] Dieval C., Deligny C., Meyer A., Cluzel P., Champtiaux N., Lefevre G., Saadoun D., Sibilia J., Pellegrin J.L., Hachulla E. (2015). Myocarditis in Patients with Antisynthetase Syndrome. Medicine.

[33] Garcia S., Martins F.R., Oliveira D., Samões B., Martins A., Terroso G., Costa L. (2022). Lymphocytic myocarditis in an overlap syndrome of systemic sclerosis and polymyositis. Clin. Rheumatol..

[34] Bergua C., Chiavelli H., Allenbach Y., Arouche-Delaperche L., Arnoult C., Bourdenet G., Jean L., Zoubairi R., Guerout N., Mahler M. (2019). In vivo pathogenicity of IgG from patients with anti-SRP or anti-HMGCR autoantibodies in immune-mediated necrotising myopathy. Ann. Rheum. Dis..

[35] Suzuki S., Nishikawa A., Kuwana M., Nishimura H., Watanabe Y., Nakahara J., Hayashi Y.K., Suzuki N., Nishino I. (2015). Inflammatory myopathy with anti-signal recognition particle antibodies: Case series of 100 patients. Orphanet J. Rare Dis..

[36] Benveniste O., Drouot L., Jouen F., Charuel J.L., Bloch-Queyrat C., Behin A., Amoura Z., Marie I., Guiguet M., Eymard B. (2011). Correlation of anti-signal recognition particle autoantibody levels with creatine kinase activity in patients with necrotizing myopathy. Arthritis Rheum..

[37] Werner J.L., Christopher-Stine L., Ghazarian S.R., Pak K.S., Kus J.E., Daya N.R., Lloyd T.E., Mammen A.L. (2012). Antibody levels correlate with creatine kinase levels and strength in anti–3-hydroxy-3-methylglutaryl-coenzyme A reductase–associated autoimmune myopathy. Arthritis Rheum..

[38] Pinal-Fernandez I., Parks C., Werner J.L., Albayda J., Paik J.J., Danoff S.K., Casciola-Rosen L., Christopher-Stine L., Mammen A.L. (2017). Longitudinal Course of Disease in a Large Cohort of Myositis Patients with Autoantibodies Recognizing the Signal Recognition Particle. Arthritis Care Res..

[39] Kao A.H., Lacomis D., Lucas M., Fertig N., Oddis C.V. (2004). Anti–signal recognition particle autoantibody in patients with and patients without idiopathic inflammatory myopathy. Arthritis Rheum..

[40] Hengstman G.J.D., Brouwer R., Vree Egberts W.T.M., Seelig H.P., Jongen P.J.H., van Venrooij W.J., van Engelen B.G. (2002). Clinical and serological characteristics of 125 Dutch myositis patients. J. Neurol..

[41] Hengstman G.J.D., ter Laak H.J., Vree Egberts W.T.M., Lundberg I.E., Moutsopoulos H.M., Vencovsky J., Doria A., Mosca M., van Venrooij W.J., van Engelen B.G. (2006). Anti-signal recognition particle autoantibodies: Marker of a necrotising myopathy. Ann. Rheum. Dis..

[42] Bandeira M., Dourado E., Melo A.T., Martins P., Fraga V., Ferraro J.L., Saraiva A., Sousa M., Parente H., Soares C. (2023). Predictors of cardiac involvement in idiopathic inflammatory myopathies. Front. Immunol..

[43] Ma X., Xu L., Li Y., Bu B. (2021). Immunotherapy reversed myopathy but not cardiomyopathy in a necrotizing autoimmune myopathy patient with positive anti-SRP and MDA-5 autoantibodies. BMC Cardiovasc. Disord..

[44] Bobirca A., Alexandru C., Musetescu A.E., Bobirca F., Florescu A.T., Constantin M., Tebeica T., Florescu A., Isac S., Bojinca M. (2022). Anti-MDA5 Amyopathic Dermatomyositis—A Diagnostic and Therapeutic Challenge. Life.

[45] Thiébaut M., Terrier B., Menacer S., Berezne A., Bussone G., Goulvestre C., Bellance R., Guillevin L., Vignaux O., Mouthon L. (2013). Antisignal Recognition Particle Antibodies–Related Cardiomyopathy. Circulation.

[46] Takeguchi-Kikuchi S., Hayasaka T., Katayama T., Kano K., Takahashi K., Saito T., Sawada J., Minoshima A., Sakamoto N., Akasaka K. (2019). Anti-signal Recognition Particle Antibody-positive Necrotizing Myopathy with Secondary Cardiomyopathy: The First Myocardial Biopsy- and Multimodal Imaging-proven Case. Intern. Med..

[47] Tanaka M., Gamou N., Shizukawa H., Tsuda E., Shimohama S. (2016). Myopericarditis in a case of anti-signal recognition particle [anti-SRP] antibody-positive myopathy. Rinsho Shinkeigaku.

[48] Vargas-Hitos J.A., Sáez-Urán L.M., Rosales-Castillo A., Jiménez-Alonso J. (2017). Constitutional syndrome and chest pain as clinical onset feature of necrotizing myopathy with myocardial involvement. Int. J. Rheum. Dis..

[49] Targoff I.N., Johnson A.E., Miller F.W. (1990). Antibody to signal recognition particle in polymyositis. Arthritis Rheum..

[50] Miller T. (2002). Myopathy with antibodies to the signal recognition particle: Clinical and pathological features. J. Neurol. Neurosurg. Psychiatry.

[51] Serban D., Spataru R.I., Vancea G., Balasescu S.A., Socea B., Tudor C., Dascalu A.M. (2020). Informed consent in all surgical specialties: From legal obligation to patient satisfaction. Rom. J. Leg. Med..

[52] Leurs A., Dubucquoi S., Machuron F., Balden M., Renaud F., Rogeau S. (2021). Extended myositis-specific and -associated antibodies profile in systemic sclerosis: A cross-sectional study. Jt. Bone Spine.

[53] Anghel D., Sîrbu C.A., Petrache O.G., Opriș-Belinski D., Negru M.M., Bojincă V.C., Pleșa C.F., Ioniță Radu F. (2023). Nailfold Videocapillaroscopy in Patients with Rheumatoid Arthritis and Psoriatic Arthropathy on ANTI-TNF-ALPHA Therapy. Diagnostics.

[54] Sambataro D., Sambataro G., Libra A., Vignigni G., Pino F., Fagone E., Fruciano M., Gili E., Pignataro F., Del Papa N. (2020). Nailfold Videocapillaroscopy Is a Useful Tool to Recognize Definite Forms of Systemic Sclerosis and Idiopathic Inflammatory Myositis in Interstitial Lung Disease Patients. Diagnostics.

[55] Bhansing K.J., Lammens M., Knaapen H.K., van Riel P.L., van Engelen B.G., Vonk M.C. (2014). Scleroderma-polymyositis overlap syndrome versus idiopathic polymyositis and systemic sclerosis: A descriptive study on clinical features and myopathology. Arthritis Res. Ther..

[56] Vonk M.C. (2021). Is there still a role for cyclophosphamide in the treatment of systemic sclerosis?. J. Scleroderma Relat. Disord..

[57] Kowal-Bielecka O., Fransen J., Avouac J., Becker M., Kulak A., Allanore Y., Distler O., Clements P., Cutolo M., Czirjak L. (2017). Update of EULAR recommendations for the treatment of systemic sclerosis. Ann. Rheum. Dis..

[58] Serban D., Smarandache A.M., Cristian D., Tudor C., Duta L., Dascalu A.M. (2020). Medical errors and patient safety culture—Shifting the healthcare paradigm in Romanian hospitals. Rom. J. Leg. Med..

[59] Morelli M.B., Bongiovanni C., Da Pra S., Miano C., Sacchi F., Lauriola M., D’Uva G. (2022). Cardiotoxicity of Anticancer Drugs: Molecular Mechanisms and Strategies for Cardioprotection. Front. Cardiovasc. Med..

[60] Martin M., Fornecker L.M., Marcellin L., Mousseaux E., Hij A., Snowden J.A., Farge D., Martin T. (2017). Acute and fatal cardiotoxicity following high-dose cyclophosphamide in a patient undergoing autologous stem cell transplantation for systemic sclerosis despite satisfactory cardiopulmonary screening. Bone Marrow Transpl..

[61] Burt R.K., Oliveira M.C., Shah S.J., Moraes D.A., Simoes B., Gheorghiade M., Schroeder J., Ruderman E., Farge D., Chai Z.J. (2013). Cardiac involvement and treatment-related mortality after non-myeloablative haemopoietic stem-cell transplantation with unselected autologous peripheral blood for patients with systemic sclerosis: A retrospective analysis. Lancet.

[62] Opinc A.H., Makowski M.A., Łukasik Z.M., Makowska J.S. (2021). Cardiovascular complications in patients with idiopathic inflammatory myopathies: Does heart matter in idiopathic inflammatory myopathies?. Heart Fail. Rev..

[63] Touma Z., Arayssi T., Kibbi L., Masri A.F. (2008). Successful treatment of cardiac involvement in dermatomyositis with rituximab. Jt. Bone Spine.

[64] Allanore Y. (2006). Effects of corticosteroids and immunosuppressors on idiopathic inflammatory myopathy related myocarditis evaluated by magnetic resonance imaging. Ann. Rheum. Dis..

[65] Riemekasten G., Opitz C., Audring H., Barthelmes H., Meyer R., Hiepe F., Burmester G.R. (1999). Beware of the heart: The multiple picture of cardiac involvement in myositis. Rheumatology.

[66] Lee Z.C., Noviani M., Yap J., Chin C.W.L., Ng S.A., Low A.H.L. (2022). Tocilizumab for systemic sclerosis with cardiac involvement: A case report. Clin. Exp. Rheumatol..

[67] Ishizaki Y., Ooka S., Doi S., Kawasaki T., Sakurai K., Mizushima M., Kiyokawa T., Takakuwa Y., Tonooka K., Kawahata K. (2021). Treatment of myocardial fibrosis in systemic sclerosis with tocilizumab. Rheumatology.

[68] Schreiber A., Elango K., Sossou C., Fakhra S., Asad S., Ahsan C. (2022). COVID-19 Induced Cardiomyopathy Successfully Treated with Tocilizumab. Case Rep. Cardiol..

